# Incidence Rate of Bee Venom Acupuncture Related Anaphylaxis: A Systematic Review

**DOI:** 10.3390/toxins14040238

**Published:** 2022-03-26

**Authors:** Seong-Hwan Ko, Hyeon-Muk Oh, Do-Young Kwon, Jae-Eun Yang, Byung-Jun Kim, Hyun-Ju Ha, Eun-Jin Lim, Min-Seok Oh, Chang-Gue Son, Eun-Jung Lee

**Affiliations:** 1 Department of Korean Rehabilitation Medicine, College of Korean Medicine, Daejeon University, Daejeon 35235, Korea; kopc95@hanmail.net (S.-H.K.); rnjseh1233@naver.com (D.-Y.K.); num4321@hanmail.net (J.-E.Y.); kimbj0912@daum.net (B.-J.K.); catscatscats@naver.com (H.-J.H.); ohmin@dju.kr (M.-S.O.); 2 Department of Korean Internal Medicine, College of Korean Medicine, Daejeon University, Daejeon 35235, Korea; oh033@naver.com; 3 Department of Integrative Medicine, Graduate School of Integrative Medicine, CHA University, 120 Haeryong-ro, Kyeong-gi, Pocheon 11160, Korea; eunjinlimsydney@gmail.com; 4 Liver and Immunology Research Center, Daejeon Korean Medicine Hospital of Daejeon University, Daejeon 35235, Korea; ckson@dju.ac.kr

**Keywords:** bee venom acupuncture, anaphylaxis, systematic review, incidence rate

## Abstract

Background: Bee venom acupuncture (BVA) is an effective treatment method for various diseases. Bee venom, however, can cause adverse effects, even rarely including life-threatening anaphylaxis, so safety-related evidence is required. In this study, we systematically estimated the incidence rate of anaphylaxis in response to BVA. Methods: We searched eight databases (MEDLINE (Pubmed), EMBASE, Cochrane Central Register of Controlled, KISS, KMBASE, Koreamed, OASIS, and NDSL) and systematically reviewed the articles that met the inclusion/exclusion criteria. Results: Among 225 potentially relevant articles, 49 were selected for this study. The overall incidence rate of anaphylaxis in response to BVA was 0.045% (95% CI 0.028–0.062). Women (0.083%, 95% CI 0.010–0.157) showed a higher incidence rate than men (0.019%, 95% CI −0.018 to 0.055), while the incidence for patients who had a skin test conducted (0.041%, 95% CI 0.011–0.072) was not significantly different compared to that obtained for patients for which there was no information about a skin test (0.047%, 95% CI 0.026–0.067). The publication year affected the incidence rate: it was highest before 1999 (1.099%, 95% CI −1.043 to 3.241), lower between 2000 and 2009 (0.049%, 95% CI 0.025–0.073), and lowest between 2010 and 2021 (0.037% 95% CI 0.014–0.060). Conclusions: In this study, we provide reference data about risk size and factors of BVA-related anaphylaxis, which is essentially required for BVA application in clinics.

## 1. Introduction

Bee venom is effective in treating pain and has recently been applied to various diseases such as arthritis, adhesive capsulitis, lupus, and cancer [1,2,3]. Bee venom acupuncture (BVA), one of the most frequently used techniques recently, involves intradermally or intramuscularly administering a small amount of refined bee venom into specific acupoints or painful areas [4].

Despite its clinical advantages, BVA often induces allergic reactions, ranging from local reactions such as rash, swelling, and itching to systemic reactions such as anaphylaxis [5]. Bee venom contains various enzymes, peptides, and biogenic amines, including melittin, phospholipase A2, and apamin [6]. Among these compounds, melittin is considered to be the main active ingredient associated with the therapeutic effect of bee venom [7]. Phospholipase A2 is known to be a major allergen that can induce anaphylaxis through inflammation and hypotension [8], even though it was also recently discovered to have immunomodulatory effects [9]. Anaphylaxis is a life-threatening systemic reaction, with clinical features such as lower blood pressure, blood-clotting tendency, and dyspnea; thus, it causes shock or seizures [10]. Therefore, it is very important to prevent anaphylaxis in clinics through the safe application of BVA.

Since the mid-2000s, in order to reduce the adverse reactions of bee venom, a method has been in use to remove its components that cause side effects, such as phospholipase A2, and extract and purify only the active ingredient, melittin, thereby creating what is called sweet bee venom (SBV) [11]. Some studies reviewing randomized controlled trials (RCTs) have reported that BVA has a higher risk of adverse events than normal acupuncture treatment, but the limitations of small-scale studies suggest a need for further research regarding the safety of BVA [12,13]. Our previous cohort study estimated a 0.047% (95% CI 0.001–0.092) incidence rate of anaphylactic reactions to BVA: there were 4 cases out of 8580 patients in one hospital over a span of 10 years [14].

Considering the impact of BVA anaphylaxis, clinic-derived data are still lacking and no comprehensive study has been conducted. We thus aimed to determine the overall incidence of anaphylaxis in response to BVA via a systematic review using all of the published literature.

## 2. Results

### 2.1. Characteristics of the Included Studies

**Figure 1:**
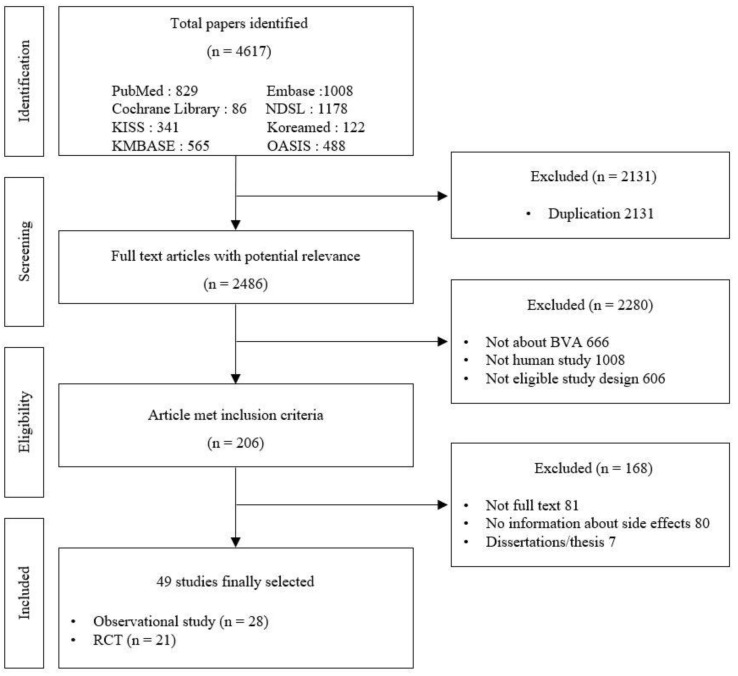
Flow diagram of the literature search process.

In total, 225 studies were identified in the initial screening, of which 49 articles (28 observational studies and 21 RCTs) met the inclusion criteria. Figure 1 shows a flow diagram of the procedure used to select the relevant studies. The characteristics of the included studies are summarized in Table 1.

The main complaints of patients who used BVA were musculoskeletal system or connective tissue disease in 31 patients, nervous system disease in 5 patients, and external causes such as injury in 3 patients. A skin test prior to BVA treatment was performed in 27 studies, while the rest did not specify whether a skin test was performed. Two studies were published between 1990 and 1999, nineteen studies between 2000 and 2009, and twenty-eight studies after 2010. These studies were from four countries: Korea (*n* = 46), France (*n* = 1), Romania (*n* = 1), and the USA (*n* = 1).

### 2.2. Incidence of Anaphylaxis in Response to BVA

**Figure 2:**
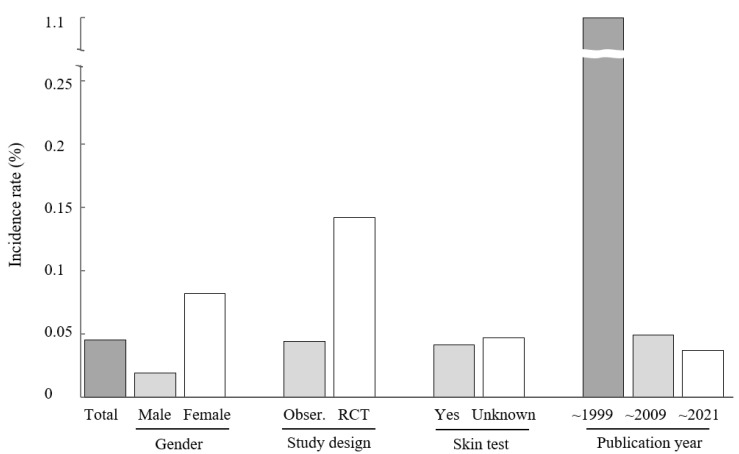
The incidence rate of anaphylaxis due to BVA by subgroup. Obser.: observational study; Unknown: no description of whether or not the skin test was performed prior to BVA treatment.

Of the total 59,733 participants treated with BVA, 27 cases of anaphylaxis were reported. The overall incidence of anaphylaxis in response to BVA was 0.045% (95% CI 0.028–0.055; Table 2 and Figure 2). The incidence was 0.044% (95% CI 0.027–0.061) in observational studies and 0.142% (95% CI −0.136 to 0.421) in RCTs.

### 2.3. Incidence of Anaphylaxis in Response to BVA by Subgroup

Thirty-seven studies describe the data by sex; women show a higher incidence rate than men (0.083% for women vs. 0.019% for men). Participants’ main complaints were available in 39 studies. Anaphylaxis was the most frequent after treatment for injury (1.299%), followed by neurological disorders (0.885%, representing one case of multiple sclerosis), and musculoskeletal disorders (0.043%). 

Performing a skin test resulted in no significant difference in the incidence of anaphylaxis compared to cases in which the performing of a skin test was not described (0.041% in skin test performed vs. 0.047% in skin test not described). We identified that the more recently the paper was published, the lower the incidence of anaphylaxis: highest before 1999 (1.099%), lower between 2000 and 2009 (0.049%), and lowest between 2010 and 2021 (0.037%) (Table 2 and Figure 2).

### 2.4. Characteristics of Patients with Anaphylaxis

Out of a total of twenty-seven cases of anaphylaxis, information about twelve patients was available (Table 3). Nine patients showed anaphylaxis at the same concentration of bee venom, 10,000:1. Venom volume at time of anaphylaxis varied from 0.1 to 2 cc. The number of treatments until anaphylaxis reported varied from 1 to 24. Of the seven available studies, two administered bee venom intradermally and five performed an intramuscular injection. Four patients were grade IV (cardiovascular symptoms such as hypotension with/without cyanosis, collapse, arrhythmias, and angina pectoris), six patients were grade III (respiratory symptoms such as dyspnea, difficulty swallowing, hoarseness, and stridor), and the rest were unknown. Except for five cases for which treatment results were not described, twenty-two cases recovered from anaphylaxis.

## 3. Discussion

In clinical fields, bee venom is used to treat pain and inflammatory symptoms because of its various properties, such as being anti-inflammatory, antibiotic, or applicable in COVID-19 treatment or prevention [15]. In general, the incidence of mild adverse reactions to BVA, such as localized edema, pruritus, and skin rash, was reported to be 28.87% [16]. However, there were case reports of two deaths due to anaphylaxis after BVA treatment [17]. Clinical use of bee venom is limited owing to these severe cases of adverse effects; however, there is no clear evidence leading to the conclusion that bee venom therapy (BVT) is not safe [18]. In fact, two large cohort studies included in our review suggested that the incidence rate of anaphylaxis in response to BVA is 0.034% and 0.019% in 32,000 and 15,783 patients, respectively [19,20]. To overcome the limitations surrounding being outdated and individual studies using a small number of subjects to calculate incidence, we systematically reviewed the incidence rate of anaphylaxis in response to BVA.

Cases of anaphylaxis after BVA treatment are reported in 8 of 49 studies, with an incidence of 0.045% (95% CI 0.028–0.062), which is consistent with our previous single-hospital retrospective studies (0.047%, 95% CI 0.001–0.092) [14].

The main causes of anaphylaxis are foods, drugs, intravenous contrast agents, and venom [21]. Nonsteroidal anti-inflammatory drugs (NSAIDs) and antibiotics such as penicillins and sulfonamides are typical medications that can induce anaphylaxis, with incidence rates of 0.130%, 0.459%, and 0.151%, respectively [22]. Compared to these conventional drugs, the incidence of anaphylaxis due to BVA does not deviate significantly from normal levels.

Regarding the study design, RCTs include only specific populations according to selection and exclusion criteria; observational studies can be more representative of incidence rates, as they include broad and random population groups [23]. In our results, the more than three fold higher incidence of anaphylaxis in RCTs (0.142%) than in observational studies (0.044%) may be due to this limitation, along with the relatively small number of participants included.

Factors influencing allergic reactions to drugs are age, sex, and family history; older people and women are more likely to develop an allergic reaction [24,25]. Although the mechanism of susceptibility to anaphylaxis is unclear, these studies support our findings that women show a higher incidence of anaphylaxis in response to BVA than men [26]. The data on main complaints and incidence of anaphylaxis show for which disease BVA was most frequently used, not which disease increases the incidence of BVA-related anaphylaxis. Out of a total of 58,448 participants with musculoskeletal and connective tissue diseases for which BVA was most frequently applied in clinics, 25 of them had a BVA-related anaphylactic reaction. Looking at the nature of the subjects’ diseases, 98% of their chief complaints were musculoskeletal disorders such as herniated nucleus pulposus, osteoarthritis, rheumatoid arthritis, etc. In addition, BVA was applied to a total of 113 patients with neurological diseases (31 patients with Parkinson’s disease, 20 with facial paralysis, 11 with peripheral neuropathy, and 51 with multiple sclerosis), of which 1 patient with multiple sclerosis had an anaphylactic reaction after BVA treatment. 

A skin test is a process performed prior to injection (for example, before antibiotic injection) to check whether an allergic reaction occurs with a small amount administered subcutaneously [27]. There is also evidence that skin tests are helpful in identifying possible anaphylaxis [28]. Surprisingly, in our study, the performance of a prior skin test did not affect the incidence rate of anaphylaxis due to BVA (0.041 vs. 0.047, Table 2 and Figure 2). Due to a lack of detailed information, the current data cannot provide evidence of a causal relationship between skin testing and incidence. We argue that the predictive ability of skin tests for BVA-induced anaphylaxis may be poorer than we expected. In our previous study, we found that 80% of patients with BVA-induced anaphylaxis had undergone a skin test or showed no reaction to several BVA treatments before anaphylaxis occurred [14]. These findings might indicate that clinicians should not rely on a skin test, but instead pay attention to other risk factors.

Along with the increase in research on the effectiveness of BVA, adverse-reaction-related studies have also been increasing recently. The reported number of anaphylaxis cases due to BVA is, fortunately, not increasing. The reason for this may be improved quality control regarding BVA and the use of BVA by well-educated physicians [29,30].

Our systematic review has some limitations. First, we must note the heterogeneity of the studies included. The studies’ participants, the control groups in RCTs, and the studies’ designs are diverse. Second, most of the studies were conducted in Korea, and so it is difficult to represent the global incidence rate. Moreover, the sample sizes used in the studies included in the analysis are too small. Lastly, there is no information on each individual patient who suffered anaphylaxis and was treated with BVA; thus, it is difficult to analyze the incidence rate according to the characteristics of each group. No details were provided regarding the dose of BVA, points, or depth of injection. Nevertheless, the major strength of this study is that it is an updated review involving the largest number of patients, to the best of our knowledge.

In conclusion, we provided physicians with comprehensive information about the rate of BVA-related anaphylaxis and its risk factors, especially its female predominance, but found no predictive ability of the skin test. However, because most of the studies included in this review were conducted in Korea, it is difficult to generalize to a global scale. Despite this limitation, our present results provide valuable reference data for clinicians and researchers looking into BVA-derived applications or developments in the future.

## 4. Materials and Methods

### 4.1. Search Strategy

A systemic literature survey was conducted using eight electronic databases: MEDLINE (Pubmed), EMBASE and Cochrane Central Register of Controlled Trials (CENTRAL), KISS, KMBASE, Koreamed, OASIS, and NDSL. Both controlled terminology (MeSH and Emtree) and free text word searching were applied. A combination of search terms and keywords included bee venom (bee venom or bee or sweet bee or honey bee or wasp bee or self-administered bee or live bee or apitoxin or bong-chim) and acupuncture (acupuncture or needle or microneedle or pharmacopuncture or inject*) and their combination. Studies published up to December 2021 were searched, with no restrictions on the publication starting point.

### 4.2. Inclusion and Exclusion Criteria

#### 4.2.1. Types of Study Design

We included observational studies and randomized controlled trials (RCTs) reporting the number of participants treated by BVA, regardless of language, and excluded those studies without full text. Types of studies such as case reports, case series, and experimental research (non-human subjects) were excluded because the incidence of BVA-related adverse events could not be estimated.

#### 4.2.2. Types of Participants

Regarding the participants, only humans who received BVA were included. There were no restrictions on age, sex, or race. 

#### 4.2.3. Types of Interventions and Comparisons

Studies using bee venom through injection or acupuncture (i.e., bee venom acupuncture and bee venom injection) for the treatment of disease were included in this review. However, bee venom immunotherapy used to desensitize allergic reactions to bee venom was excluded. Live bee stings, propolis, and bee venom creams were excluded. Studies with other interventions (e.g., acupuncture, herbal medicine, massage, exercise, etc.) combined with BVA were included to confirm and estimate the incidence of BVA-related anaphylaxis. There were no restrictions on the comparison.

#### 4.2.4. Type of Outcome Measures

Studies that did not report the presence or absence of BVA-related side effects were excluded, regardless of other outcome measures. 

### 4.3. Data Extraction and Review Process

We extracted data on the number of participants, mean age, country, publication year, number of anaphylaxes, main complaints according to the international classification of diseases (ICD-10) [31], and whether a skin test was performed. The overall incidence and the incidence by subgroup were investigated. For each case of anaphylaxis, we specified the concentration and volume at the time of anaphylaxis, the cumulative number of treatments until anaphylaxis, administration method, anaphylaxis grade according to Mueller’s classification method [32], and treatment results.

Two reviewers (SHK and HMO) independently searched the titles and abstracts of the search results. Discrepancies were discussed by the two researchers, and when necessary, with a third reviewer (EJL). Potentially relevant articles not meeting all of the necessary inclusion criteria were excluded from this study. The incidence rate was determined by calculating the number of anaphylaxis occurrences among the total number of subjects.

### 4.4. Statistical Analysis

The pooled incidence rate is given as a percentage within the 95% confidence interval. Categorical variables (number of participants and studies) were analyzed as frequency and percentages by frequency analysis, and averages for the ages of total and subgroup participants are expressed as the means ± standard deviation (SD). All statistical analyses were performed with the SPSS statistical software package version 28.0 (SPSS Inc., Chicago, IL, USA).

## Figures and Tables

**Table 1:** Characteristics of included studies.

Items	Observational Studies	RCTs	Total
Number of studies (%)	28 (57)	21 (43)	49 (100)
Number of participants (%)	59,030 (99)	703 (1)	59,733 (100)
Men/Women	5227 (48)/5739 (52)	176 (33)/371 (67)	5403/6110
No information ^a^	48,064	156	48,220
Mean age (years) ^b^	47.5 ± 8.6	55.5 ± 6.83	51.1 ± 8.8
Main complaints (No. of participants, studies)			
G00–G99 ^c^	93 (4)	20 (1)	113 (5)
S00–T98 ^d^	16 (1)	61 (2)	77 (3)
M00–M99 ^e^	57,897 (15)	551 (16)	58,448 (31)
Others (obesity)	0 (0)	51 (1)	51 (1)
No information ^a^	1024 (8)	20 (1)	1044 (9)
Skin test (No. of participants, studies)			
Yes	16,585 (14)	375 (13)	16,960 (27)
No	0 (0)	0 (0)	0 (0)
No information ^a^	42,445 (14)	328 (8)	42,773 (22)
Publication year (No. of participants, studies)			
–1999	31 (1)	60 (1)	91 (2)
2000–2009	32,350 (10)	409 (9)	32,759 (19)
2010–2021	26,649 (17)	234 (11)	26,883 (28)
Country (No. of participants, studies)			
South Korea	58,953 (26)	683 (20)	59,636 (46)
France	0 (0)	20 (1)	20 (1)
Romania	26 (1)	0 (0)	26 (1)
USA	51 (1)	0 (0)	51 (1)

^a^ The relevant information is not present in the articles; ^b^ mean age is the average of studies that presented the mean age of participants; ^c^ diseases of the nervous system; ^d^ injury, poisoning, and certain other consequences of external causes; ^e^ diseases of the musculoskeletal system and connective tissue.

**Table 2:** Incidence of anaphylaxis due to BVA by subgroup.

Group	Incidence (%, 95% CI)		
	Observational Studies (59,030 Participants)	RCTs (703 Participants)	Total (59,733 Participants)
No. of anaphylaxis	26	1	27
Overall incidence in all studies	0.044 (0.027–0.061)	0.142 (−0.136 to 0.421)	0.045 (0.028–0.062)
Incidence by subgroup (No. studies that presented data)			
Sex (37)			
Men	0.019 (−0.019 to 0.057)	0	0.019 (−0.018 to 0.055)
Women	0.089 (0.011–0.167)	0	0.083 (0.010–0.157)
Main complaints (39)			
G00-G99 ^a^	1.075 (−1.021 to 3.171)	0	0.885 (−0.842 to 2.612)
S00-T98 ^b^	0	1.639 (−1.547 to 4.826)	1.299 (−1.230 to 3.828)
M00-M99 ^c^	0.043 (0.026–0.060)	0	0.043 (0.026–0.060)
Others (obesity)	0	0	0
No information ^d^	0	0	0
Skin test (49)			
Yes	0.036 (0.007–0.065)	0.267 (−0.255 to 0.789)	0.041 (0.011–0.072)
No			
No information ^d^	0.047 (0.026–0.068)	0	0.047 (0.026–0.067)
Publication year (49)			
–1999	3.226 (−2.994 to 9.446)	0	1.099 (−1.043 to 3.241)
2000–2009	0.049 (0.025–0.074)	0	0.049 (0.025–0.073)
2010–2021	0.034 (0.012–0.056)	0.379 (−0.362 to 1.120)	0.037 (0.014–0.060)

^a^ Diseases of the nervous system; ^b^ injury, poisoning, and certain other consequences of external causes; ^c^ diseases of the musculoskeletal system and connective tissue; ^d^ relevant information is not presented in the articles.

**Table 3:** Characteristics of anaphylaxis cases (27 cases).

Patient Number	Sex/Age	Concentration during Anaphylaxis	Volume during Anaphylaxis	No. Treatments until Anaphylaxis	Administration	Anaphylaxis Grade ^c^	Treatment Result
P1	Unknown	Unknown	Unknown	4	Unknown	III	Recovered
P2–5	4 patients /(Unknown)	Unknown	Unknown	Unknown	Unknown	Unknown	Unknown
P6–16 ^a^	11 patients /(40.9 ± 12 ^a^)	Unknown	1.6 cc ± 0.5	10.9 ± 6.8	Unknown	Unknown	Recovered
P17 ^b^	Unknown	Unknown	0.4 cc	24	Intradermal	Unknown	Unknown
P18	Unknown	10,000:1	Unknown	Unknown	Unknown	IV	Recovered
P19	F/36	Unknown	2 cc	6	Intradermal	Unknown	Recovered
P20	F/61	10,000:1	0.5 cc	1	Unknown	III	Recovered
P21	F/65	10,000:1	0.1 cc	Test	Unknown	III	Recovered
P22	F/77	10,000:1	1 cc	Unknown	Unknown	IV	Recovered
P23	M/70	10,000:1	2 cc	6	Intramuscular	III	Recovered
P24	F/59	10,000:1	2 cc	2	Intramuscular	IV	Recovered
P25	F/62	10,000:1	2 cc	13	Intramuscular	III	Recovered
P26	F/60	10,000:1	2 cc	6	Intramuscular	III	Recovered
P27	F/42	10,000:1	2 cc	22	Intramuscular	IV	Recovered

^a^ All items are the mean values of 11 patients, ^b^ volume and treatment session are estimated from data presented, **^c^** Mueller grade.

## Data Availability

The data used for this study are available from the corresponding author upon request.
